# Metals and Alloys

**Published:** 1863-02

**Authors:** B. Wood

**Affiliations:** New York


### METALS AND ALLOYS.

BY DR. B. WOOD.

(Continued from page 536, Vol. XVI.)

### ANTIMONY.

Hardness 7. Flex. 1. Mall. 1. Fus. 8.

Antimony is a hard, brittle metal, of a brilliant silver-white color, with a light bluish tint. With the usual impurities, as obtained in commerce, it is of a gray bluish white. When slightly tarnished from exposure to the atmosphere, the surface assumes a dark gray color with a greenish tinge, and here and there iridescent gleams of lilac or purple. Its structure is crystalline and lamellated. When broken, the fracture reveals bright glistening crystals, resembling those of bismuth, but having smaller facets, and a silvery white color. Though sometimes mistaken for bismuth, it is readily distinguished by this crystalline appearance, as well as by its hardness. It breaks more suddenly, and with a snap, while bismuth yields rather gradually, breaking with a sort of tear, and a creaking sound. Bismuth may be slightly reduced under the hammer before breaking. Antimony not.

Its melting point is variously stated from 797° to 1000°, being usually put at 800° Fahr. It melts at a fair red heat some where between the melting point of zinc and of silver but nearer to that of the former, and about the same as aluminium.

At a read heat, exposed to the air, it burns with a bluish white flame, emitting white fumes of oxide. Fused with charcoal and thrown suddenly on the ground, the divided globules burn vividly, emitting brilliant sparks. It may, by exclusion of air, be distilled at a white heat. It exibits a strong galvanic action with gold and silver, and less with platinum.

Although it is said air has no effect upon it, it nevertheless tarnishes in the atmosphere, but less readily than lead, bismuth, cadmium or tin. Water is not decomposed by it. Caustic potash has no action upon it. The alkaline sulphurets are said to dissolve it completely. Tartaric acid attacks it slowly. Sulphuric acid more promptly but with little energy. The action of hydrochloric acid is very feeble when cold, but heated to boiling it dissolves it. Nitric acid in the cold state oxidizes it, rather slowly, but when heated the action is energetic with the evolution of gas bubbles in the form of small needle-shaped jets, like miniature water spouts. It is not dissolved by this action, but converted into peroxide, called, owing to its property of combining with bases, antimonic acid, which is precipitated from the acid in the form of a light yellow powder. This oxide, or acid, precipitated from nitric acid, is soluble in boiling solution of tartaric acid, while the oxide of tin, or stannic acid, precipitated from the same menstruum, is not. By this means they may be distinguished and separated, which it is well to note, having omitted to mention it when speaking of the process of purifying tin.

The sulphuret of antimony passes in commerce under the name of crude antimony, or simply antimony, while the metal is called *Regulus* of Antimony.

The sulphuret was formerly employed in the preparation of alloys. It is of a leaden gray-color, very brittle, and much more fusible than the metal. It is desulphurized and reduced to the metallic state by first roasting it, by which it is oxidized and most of the sulphur dispelled, and then subjecting the oxide mixed with half its weight of tartar, to a high red heat; or it may be mixed with carbonate of soda and charcoal, reduced to powder, or with charcoal impregnated with a strong solution of carbonate of soda. The alkali combines with the remaining sulphur, forming with it a flux or scoria, while the charcoal reduces the oxide to an impure "regulus," which is further pacified by a second fusion with a small proportion of oxide of antimony. The regulus, as obtained in commerce, may also be freed from most of its impurities by mixing the powdered metal with one-tenth of its weight of nitric and fusing in a clay or earthen crucible. By this means the impurities are oxidized and held in the scoria while the refined metal sinks to the bottom. For more exact results recourse must be had to the hurried process (by solution in acids, etc.) We omit this to save space, as these papers are already extended beyond the original intention, and since absolute purity is not essential for the uses to which this metal is likely to be put. Indeed we may hereafter omit this part of the subject, as foreign to the topic, not designing to treat of the chemistry of metals except so far as aiding to unfold the characteristics and relations of metals in respect to each other, as in the formation of alloy, etc. Besides it is really of little practical use to treat upon these points without going into the details, to do which would require a separate paper for each metal.

Antimony alloyed with other metals renders them hard and brittle, confers greater brilliancy, and serves to counteract shrinkage. The more oxidable metals when alloyed with it are generally less susceptible to oxidation than they were before. The prevalent theory that metals are more oxidable when alloyed with others, like many other theories based on a few particular instances, is true in some cases, in others not. Some alloys are more oxidable than the most oxidable of their constituents, but many alloys resist oxidation as well as the least oxidable metal that enters into the compound, and some even better. But no "new metal" can be formed by alloying that will resist the action of any agent capable of dissolving or oxidating each of its components separately, although it might be able to withstand the common influences to which it was liable to be subjected as effectually as any one of them, or even more effectually.

With tin, antimony forms alloys which remain remarkably clean and bright. It combines so intimately with tin that it is not separated to any appreciable extent by the heat sufficient to volatilize it by itself or in other combinations, and the alloy is not easily acted upon by any weak acid. Hence the poisonous effects of the metal are not liable to supervene from its use in small proportion in pewter wares, of which it is always a component. In large proportion it renders tin hard and brittle, but one-tenth part or less does not essentially impair its tenacity and malleability.

It renders lead brittle where used even in small proportion, in which it contrasts with bismuth; lead alloyed with one-tenth or one-twelfth part of antimony corresponding in this quality with an alloy consisting of equal parts of lead and bismuth, both being brittle and semi-malleable; while one-tenth part of bismuth does not at all impair the tenacity of lead. In this particular antimony bears a relation to lead similar to that of bismuth to tin, a like proportion of each producing a like result in each respective case. In moderate proportion it somewhat increases the fusibility of lead, but diminishes that of tin, bismuth, cadmium and zinc. Its alloy with bismuth is hard and very brittle, exhibits, when burnished, prismatic hues, and when broken a brilliant crystalline fracture. With cadmium and zinc it forms still harder alloys, which are also very brittle. Tin causes it to unite more kindly with either of these metals and the ternary compounds thus produced are valuable for their hardness and strength. The same holds in respect to the compounds of antimony, tin and copper, although these, with a like proportion of tin are brittler than the former. They all furnish alloys, when the ingredients are used in suitable proportions, which shrink very little, if at all, in casting.

Its affinity for arsenic appears to be strong, and so intimate the union, that the latter metal is said to enter into most of the antimonial preparations. "According to Serullees, all the antimonial preparations, except tartar emetic, and butter or sesquichloride of antimony, contain a minute portion of arsenic. Tartar emetic is an exception, because, according to this chemist, it separates entirely, in the act of crystallizing, from any minute portion of arsenic in the materials from which it is prepared; the poisonous metal being left behind in the mother-waters of the process." (*U. S. Dispensatory.*) In this respect it contrasts with bismuth which has a weak affinity for arsenic, and may be effectually freed from it by proper care. "Where gold is alloyed with a two hundreth part of antimony, the compound is brittle; and even the fumes of antimony in the vicinity of melted gold are sufficient to render it brittle."—*Brande.*

Several preparations of antimony are employed medicinally. The princ'ple and most important form used is the tartrate of antimony and potassa, or tartar emetics. "Its general action is that of a sedative upon the circulation, while, on the contrary, it excites most of the secretions. According to the dose, and the peculiar circumstances under which it is administered, it acts variously, as an alterative, diaphoretic, diuretic, expectorant, purgative, and emetic. (*U. S. Dispensatory.*) Externally it is used as a counter-irritant, producing a characteristic pustular eruption, attended with a deep, gnawing, burning *torment.*

In the arts, antimony forms an important constituent in type metal (for which almost every author gives a different formula) in stereotype metal, in britannia metal, queen's metal and other forms of pewter ware.

It constitutes, according to the specification, an essential ingredient in Dr. Blandy's cheoplastic metal for casting artificial dentures. In mechanical dentistry it is used to some extent in the formation of dyes. An alloy for this purpose consisting of one part antimony to five parts of tin was announced a few years ago as an important discovery. It requires particular manipulation when cast in sand, to prevent fissures and shrinkage on the face of the die. A better alloy, and free from this difficulty may be formed by combining one part of antimony, one part zinc and sixteen parts of tin.

The next number will treat of lead and zinc, after which we will proceed to particularize alloys.

*New York, January,* 1863.

